# Leveraging the Adult Wellbeing CheckUp Platform to Enhance Social Connection and Well-Being Among Older Adults

**DOI:** 10.1093/geroni/igaf122.1333

**Published:** 2025-12-31

**Authors:** Yong Kyung Choi, Diana Yin, Ronald Kaiser, James Firman

**Affiliations:** University of Pittsburgh, Pittsburgh, Pennsylvania, United States; BetterAge, Sunnyside, New York, United States; BetterAge, Sunnyside, New York, United States; BetterAge, Arlington, Virginia, United States

## Abstract

The Adult Wellbeing CheckUp (AWC) is an innovative web-based platform, designed and built by BetterAge, to assess older adults’ well-being and deliver personalized recommendations to enhance health, social engagement, and quality of life. Supported by the National Institute on Aging (NIA) SBIR program, AWC integrates a multi-domain assessment covering physical, mental, and social health, generating customized action plans based on individual priorities. The platform applies behavioral change principles, curated peer support, and local service referrals to help older adults, including care partners (CPs), take actionable steps toward well-being. AWC was developed using a user-centered design approach through three focus groups (N = 77; 40 general older adults (OAs), 37 older adult CPs) across senior centers in PA, MA, and TX. Findings revealed key differences in well-being challenges and technology use. Social isolation was a major concern among OAs, with participants citing limited engagement opportunities, mobility barriers, and difficulty accessing meaningful activities. While many relied on family or close friends, they expressed a need for structured support and community engagement. CPs, who also experience social isolation, highlighted emotional exhaustion, time constraints, and lack of self-care due to caregiving demands. Phase I pilot testing (N = 48; 24 OAs, 24 CPs) is currently underway and involves four weeks of engagement with AWC. Preliminary findings suggest high acceptability, as participants value tailored recommendations and ease of navigation. These findings underscore the importance of digital tools in addressing the unique well-being needs of OAs and CPs, particularly in reducing social isolation and supporting self-care.

